# Goreisan attenuates cardiac hypertrophy and diastolic dysfunction in heart failure with preserved ejection fraction induced by HFD/L-NAME via regulation of ICAT-β-catenin/ERK axis

**DOI:** 10.1038/s41440-025-02348-z

**Published:** 2025-09-03

**Authors:** Yoko Shojima Isayama, Shouji Matsushima, Keisuke Shinohara, Koichi Isayama, Nobuyuki Enzan, Taishi Yamamoto, Masashi Sada, Ryo Miyake, Yoshitomo Tsutsui, Takayuki Toyohara, Ryohei Nishimura, Yuki Ikeda, Eri Noda, Wataru Otsuru, Shuya Tokumoto, Masatsugu Watanabe, Masataka Ikeda, Toru Hashimoto, Shintaro Kinugawa, Hiroyuki Tsutsui, Kohtaro Abe

**Affiliations:** 1 Department of Cardiovascular Medicine, Faculty of Medical Sciences, Kyushu University, Fukuoka, Japan; 2 Division of Cardiovascular Medicine, Research Institute of Angiocardiology, Faculty of Medical Sciences, Kyushu University, Fukuoka, Japan; 3 Department of Anesthesiology and Critical Care Medicine, Graduate School of Medical Sciences, Kyushu University, Fukuoka, Japan; 4 School of Medicine and Graduate School, International University of Health and Welfare, Fukuoka, Japan

**Keywords:** Goreisan, heart failure with preserved ejection fraction, cardiac hypertrophy, diastolic dysfunction

## Abstract

Heart failure with preserved ejection fraction (HFpEF), characterized by cardiac hypertrophy and diastolic dysfunction, is increasing worldwide. Goreisan (GRS) is a traditional herbal formulation; its component attenuates cardiomyocyte hypertrophy. This study aimed to investigate the effect of GRS on the pathophysiology of HFpEF. Administration of a high fat diet (HFD, 60% fat) and N-nitro-L-arginine methylester (L-NAME, 0.5 g/L in drinking water) increased heart and lung weights in C57BL/6 mice and GRS (5.9 mg/kcal) reduced them without changes in blood pressure. GRS attenuated HFD/L-NAME-induced increases in left ventricular wall thickness and E/A and E/E’, indices of diastolic dysfunction. GRS decreased cardiomyocyte cross-sectional area in HFD/L-NAME-treated mice. Mechanistically, it suppressed the phosphorylation of mitogen-activated protein kinases (MAPKs), such as extracellular signal-regulated kinase (ERK), in HFD/L-NAME-treated hearts. In addition, liquid chromatography/mass spectrometry demonstrated that HFD/L-NAME decreased and GRS increased 73 proteins in the heart. Among them, GRS prevented HFD/L-NAME-induced decrease in inhibitor of β-catenin and T-cell factor (ICAT), a negative regulator of cardiac hypertrophy. Consistently, β-catenin, an ICAT target, exhibited the opposite change. In in vitro experiments, GRS directly decreased β-catenin in isoproterenol (ISO)-treated cardiomyocytes, accompanied by a decrease in cardiomyocyte surface area. Overexpression of ICAT also suppressed ISO-induced increases in β-catenin, phosphorylated ERK, and cardiomyocyte surface area. Among GRS ingredients, cinnamaldehyde and alisol B 23-acetate attenuated ISO-induced increases in β-catenin and cardiomyocyte surface area. In conclusion, GRS attenuates cardiac hypertrophy and diastolic dysfunction via ICAT-β-catenin/ERK axis. GRS is a potential herbal formulation for the treatment of HFpEF.

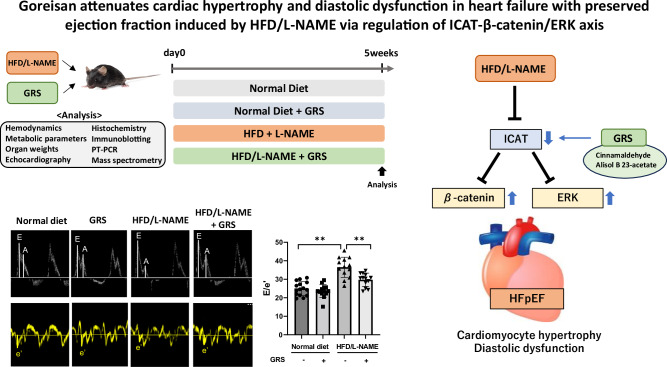

## Introduction

Heart failure (HF) is a major public health issue worldwide [1]. By 2030 and 2035, the number of patients with HF is expected to increase to 8 million in United States [2] and 1.3 million in Japan [3], respectively. Approximately half of all patients with HF have HF with preserved ejection fraction (HFpEF) and its mortality rate is comparable with that of HF with reduced ejection fraction (HFrEF) [4, 5]. Cardiac hypertrophy and diastolic dysfunction are the fundamental components underlying the pathophysiology of HFpEF [6]. Although sodium-glucose cotransporter 2 (SGLT2) inhibitors are effective for HFpEF, [7, 8] the therapeutic strategy for HFpEF, especially diastolic dysfunction, has not been fully established.

Recently, a mouse HFpEF model created by administrating a high fat diet (HFD) and N-nitro-L-arginine methyl ester (L-NAME), known as the two hits model, has been reported [9]. This model mimics the pathophysiology of human HFpEF, including pulmonary congestion/edema, cardiac hypertrophy, and diastolic dysfunction and is useful for experimental research on HFpEF. In this model, endoplasmic reticulum (ER) stress and inflammation, in addition to mitogen-activated protein kinases (MAPKs), are involved in the HFpEF pathophysiology [9–11].

Goreisan (GRS) is a traditional herbal formulation with diuretic properties, which is widely used for the treatment of edema in Japan [12]. It is specifically used for the treatment of patients with chronic subdural hematoma [13]. Aquaporin 2 is speculated to mediate the water-regulating effect in the kidneys [14, 15]. Several recent studies have reported the potential efficacy of GRS on cardiovascular diseases. In some patients with HF, GRS is effective for decongestion [15, 16]. Furthermore, cinnamaldehyde, a component of GRS alleviates pressure overload- or phenylephrine-induced cardiac hypertrophy [17–19]. In addition, GRS has anti-inflammatory effects on glomerulosclerosis [20] and lipopolysaccharide-mediated inflammation [21]. These findings suggest that GRS might be beneficial for the treatment of HFpEF. Clinically, the first large-scale randomized trial regarding the impact of GRS on HF is underway [22]. Thus, GRS is highlighted in the field of HF. However, the precise effect of GRS on HFpEF and its biological mechanism remain unclear.

This study aimed to determine whether GRS attenuates the pathophysiology of HFpEF and elucidate the mechanism mediating its beneficial effects using a mouse model of HFpEF.

## Methods

### Drug reagents

Powdered extracts of GRS (lot no. 2210017020) were supplied by Tsumura and Co. (Tokyo, Japan). GRS is a mixture of five constituent crude drugs: four parts Alisma Tuber (tuber of *Alisma orientale* Juzepczuk), three parts Atractylodes Lancea Rhizome (rhizome of *Atractylodes lanceae* De Candolle), three parts Polyporus Sclerotium (sclerotium of *Polyporus umbellatus* Fries), three parts Poria Sclerotium (sclerotium of *Poria cocos* Wolf), and one and a half part of Cinnamon Bark (*Cinnamomum cassia* J. Presl). A mixture of the five crude drugs was extracted using purified water at 100 °C for 1 h. Spray drying was used to dry the extraction. GRS quality was standardized and guaranteed by measuring a characteristic marker (i.e. (E)-cinnamic acid) based on good manufacturing practices in Japan. L-NAME and isoproterenol (ISO) were purchased from Sigma-Aldrich (St. Louis, MO, USA) and Tokyo Chemical Industry Co., Ltd (Tokyo, Japan), respectively.

Cinnamaldehyde was purchased from Sigma-Aldrich (St. Louis, MO, USA). Cinnamic acid, alisol B 23-acetate, and atractylodin were purchased from Tokyo Chemical Industry Co., Ltd. (Tokyo, Japan). Pachymic acid was obtained from Toronto Research Chemicals Inc. (North York, ON, Canada).

### Animal experiments

Male C57BL/6 J mice (9–10 weeks old, 20–25 g) were purchased from The Jackson Laboratory Japan, Inc. (Kanagawa, Japan). The mice were housed under a 12-h/12-h light-dark cycle at 23 °C ± 3.0 °C and humidity of 50% ± 20%. They were allowed free access to water and standard laboratory feed. Normal diet (D12450J) and normal diet containing GRS (5.9 mg/kcal), HFD (D12492), and HFD containing GRS (5.9 mg/kcal) were purchased from Research Diet Inc. (New Brunswick, NJ, USA). The mice were divided into four treatment groups (Supplementary Fig. 1). We determined administrative doses of GRS for in vivo and in vitro experiments based on previous studies [23]. In addition, the mixture ratio (3%) for administering GRS mixed feed for the HFD/L-NAME + GRS group was set based on a previous paper [24]. Furthermore, the dosage for the normal diet + GRS group was designed to be the same as that for the HFD/L-NAME + GRS group in terms of calorie content.

All experimental procedures were conducted by the Guideline for Animal Experiments of Kyushu University and the Care. All the experimental procedures were approved by the Committee on Ethics of Animal Experiments of the Kyushu University Graduate School of Medicine and Pharmaceutical Sciences (A21-381). Furthermore, the procedures met the ethical standards required by the laws and guidelines for experimental animals in Japan. This study was conducted and reported in accordance with the ARRIVE guidelines.

### Primary culture of neonatal rat ventricular myocytes

Primary cultures of neonatal rat ventricular myocytes (NRVMs) were prepared from 1-day-old Wister rats (Kyudo Inc, Japan). A cardiac myocyte-rich fraction was obtained by centrifugation through a discontinuous Percoll gradient as previously described [25]. NRVMs were treated with ISO (10 µM, 48 h) and GRS (100 μg/ml, 48 h). We used phosphate-buffered saline (PBS) for ISO and dimethyl sulfoxide (DMSO) for GRS as vehicle controls with a final volume concentration of 0.1%.

### Quantitation of cell surface area of NRVMs

NRVMs grown in glass plate dish were washed 3 times with PBS. The cells were fixed with 4% paraformaldehyde for 15 min and washed 3 times with PBS. The cells were then permeabilized with 0.1% Triton X-100 for 15 min and washed 3 times with PBS. The cells were incubated with blocking buffer (1% BSA in PBS) for 1 hour. Subsequently, the NRVM_S_ were incubated with a Phalloidin (Green Fluorescent Conjugate, Acti-stain 488, Cytoskeleton, Inc.) at room temperature in the dark for thirty minutes and then washed 3 times with PBS. The cells then mounted with VECTASHIELD mounting medium with DAPI (H-1200, Vector Laboratories). Pictures of cardiomyocytes were obtained with a fluorescent microscope (BZ-X800, Keyence). Cell surface area of 100 cardiomyocytes randomly selected from five dishes in each group was evaluated with the BZ- X800 Analyzer (1.1.1.8) software.

### Plasmids and viral vector construction

The adenoviral vectors used to overexpress ICAT-His-tag were constructed and packaged by VectorBuilder. The vector ID is VB240204-1362jqq respectively, which can be used to retrieve detailed information about the vector on vectorbuilder.com.

### Measurement of blood pressure and heart rate

As previously reported, a noninvasive tail-cuff system was used to measure blood pressure and heart rate (Model MK-1030 NIBP Monitor for Rats & Mice) [26].

### Measurement of the metabolic parameters

Blood glucose levels were measured using FreeStyle Freedom^TM^ Lite (Abbott Japan CO., LTD. Tokyo, Japan, 70959-70). Plasma total cholesterol levels in peripheral blood samples were measured using LabAssay TM cholesterol (FUJIFILM Wako Pure Chemical Corporation. Osaka, Japan, 294-65801).

### Evaluation of extracellular volume (ECV), plasma volume (PV), and interstitial fluid volume (IFV)

Mice were administered Evans blue (50 μg) and D-mannitol (5 mg) through the tail vein. Plasma was obtained after 10 min. The concentration of Evans blue or D-mannitol in the plasma was determined by measuring absorbance at 620 nm or using D-mannitol colorimetric assay kit (Sigma-Aldrich, MO, USA, MAK514). ECV and PV were calculated using dosage/concentration. IFV was calculated as ECV–PV.

### Echocardiography

Under light anesthesia with 1-2% isoflurane, two-dimensional targeted M-mode images were obtained from the short axis view at the papillary muscle level using a Vevo 2100 ultrasonography system (Visual Sonics, Toronto, Canada) as previously described [27]. LVEF = [(diastolic LV volume – systolic LV volume)/diastolic LV volume) × 100], where LV volume = [(7.0 / (2.4  +  LV diameter)] × LV diameter. Subsequently, diastolic function (E wave, A wave, and e’) was assessed at the mitral valve level using pulse wave and tissue Doppler imaging. Anesthesia was maintained at 2% isoflurane and adjusted to keep a heart rate of approximately 400. LV wall thickness was calculated as the average of interventricular septum and posterior wall thicknesses.

### Histological analyses

The LV was cut into base and apex portions, fixed with 10% formalin, and stained using hematoxylin and eosin. Mid-LV staining with wheat germ agglutinin (WGA) was used to evaluate the myocyte cross-sectional area, as described previously [28]. Collagen volume was determined by quantitative morphometry of tissue sections from the mid-LV stained with Masson’s trichrome, as described previously [29].

### Immunoblot analyses

Heart homogenates and cardiomyocyte lysates were prepared in RIPA lysis buffer containing Tris-HCl, NaCl, NP-40, sodium deoxycholate, and sodium dodecyl sulfate. For immunoblot analyses, we used antibodies against phosphorylation of extracellular signal-regulated kinase (p-ERK) (1:5000, CST #9101), ERK (1:5000, CST #9102), c-Jun N-terminal kinase (JNK) (1:5000, CST #9252), phosphorylation-p38 (p-p38) (1:5000, CST #4511), p38 (1:5000, CST #9212), phosphorylation-Akt (p-Akt) (Ser473) (1:5000, CST #4060), Akt (1:5000, CST #4691), phosphorylation-nuclear factor κB (1:5000, p-NFκB) (Ser536) (CST #3033), NFκB (1:5000, CST #8242), PERK (1:5000, CST #3192), CHOP (1:5000, CST #5554), ICAT (1:1000, sc-293489), β-catenin (1:5000, sc-7963), and glyceraldehyde-3-phosphate dehydrogenase (GAPDH) (1:20000, sc32233). GAPDH and Coomassie Brilliant Blue (CCB) staining were used as a loading control.

### Quantitative real-time PCR reaction

Quantitative real-time (RT)-PCR has been previously described [30]. ISOSPIN Cell & Tissue RNA (Nippon Gene, Japan) was used to extract total RNA. RNA was converted to cDNA using a TOYOBO ReverTra Ace qPCR RT kit. The reactions were run in an Applied Biosystems QuantStudio3 (Thermo Fisher Scientific) for the THUNDERBIRD SYBR qPCR Mix (TOYOBO, Tokyo, Japan). We used the following oligonucleotide primers in the SYBR qPCR: Rps18, sense 5′-TTCTGGCCAACGGTCTAGACAAC-3′ and antisense 5′-CCAGTGGTCTTGGTGTGCTGA-3′; TNFα sense 5′-ATCGGTCCCCAAAGGGATGA-3′ and antisense 5′-GGTGGTTTGCTACGACGTG-3′; IL-1β sense 5′-TCCAGGATGAGGACA-3′ and antisense 5′-GAACGTCACACACCACAGGTTA-3′; NOS2 sense 5′-GTTCTCAGCCCAACAATACAAGA-3′ and antisense 5′-GTGGACGGGTCGATGTCAC-3′; XBP1s sense 5′-GGTCTGCTGAGTCCGCAGCAGG-3′ and antisense 5′-GAAAGGGAGGCTGGTAAGGAAC3′. The transcripts were normalized to Rps18.

### LC-MS/MS and data processing for proteomics

The hearts were homogenized in RIPA buffer. Proteins were precipitated using 10 times volume of acetonitrile (FUJIFILM Wako Pure Chemical Corporation), redissolved with 0.5% (w/v) sodium deoxycholate (Sigma-Aldrich) in 250 mM ammonium hydrogen carbonate (Nacalai tesque, INC., Kyoto, Japan) buffer, reduced with 5 mM dithiothreitol (Sigma-Aldrich) at 60 °C for 30 min, and alkylated with 15 mM iodoacetamide (Sigma-Aldrich) at room temperature in the dark for 10 min. Following five-fold dilution with water, the solutions were added to trypsin (modified sequence grade, Promega, Madison, WI) and incubated at 37 °C overnight. After deoxycholic acid removal with ethyl acetate (Kanto Chemical Co. Inc., Tokyo, Japan) extraction at pH 2, the peptides were desalted using Stage tips with Empore disk SDB-XC (GL Science, Tokyo, Japan).

Each sample underwent Nano LC-MS/MS. The peptides were separated on an Easy-nLC1200 system (Thermo Fisher Scientific) using an EASY-Spray LC column ES905 (2 μm, 0.075 × 750 mm). The mobile phase flow rate was 200 nL/min, consisting of 0.1% formic acid in water (A), 0.1% formic acid (FUJIFILM Wako Pure Chemical Corporation), and 80% acetonitrile (B). The gradient profile was set as follows: 2%–35% B for 151 min, 35%–95% B for 2 min, and 95% B for 12 min. MS analysis was performed using Orbitrap ID-X (Thermo Fisher Scientific) with the FAIMS-Pro Interface. FAIMS switched between CVs of −40, −60, and −80 V with a cycle of 1 s. The spray voltage was set to 2 kV. Orbitrap FTMS full scan spectra were collected from 375–1500 m/z at a resolution of 60,000 followed by a data-dependent HCD ITMS product ion scan using a collision energy of 30% and an isolation width of 1.6 Da.

Protein identification and quantification were performed using Proteome Discoverer (version 2.5.0.400) with the label-free quantification workflow. The protein sequence database was combined with the UniProt reference proteome of Mus musculus (25,390 sequences, downloaded on 09 Mar 2022) and the common contaminants database from Thermo Fisher Scientific (PD_Contaminats_2015_5.fasta, 298 sequences, last modified). The search engine was Sequest HT and MS Amanda 2.0. The max trypsin miss cleavage was 2; MS1 and MS2 tolerances were 10 ppm and 0.4 Da, respectively. Static modification was carbamidomethylation of cysteine and dynamic modification was methionine oxidation, peptide N-terminal pyroglutamine, and protein N-terminal acetylation. A false discovery rate of 1% was applied to the results. We filtered proteins unlabeled as “Master Protein”.

The abundance ratios of proteins were normalized to rectify unequal protein content. The P-values were calculated using a two-tailed t-test and adjusted using the Benjamini-Hochberg method. Differently expressed proteins (DEPs) were defined as *P* = 0.05 with an abundance ratio greater than 1.25 or less than 0.8. GO enrichment analyses for DEPs were performed using DAVID (https://david.ncifcrf.gov, version 2023q4).

Proteomic files were deposited at the Proteome Xchange36 Consortium via the JPOST partner repository (https://repository.jpostdb.org) under the identifiers PXD049259 for ProteomeXchange and JPST002502 for jPOST.

### Statistical analysis

All values were expressed as mean ± standard error (SE). The Shapiro-Wilk test was used to confirm data normality. Comparisons between two groups were performed using Student’s t-test, whereas comparisons among more than two groups were conducted using analysis of variance with Tukey’s post hoc test for multiple pairwise comparisons. The t-test (Benjamini-Hochberg method) was used for adjusted *P*-value in mass spectrometric analysis. Statistical significance was determined at *P* < 0.05.

## Results

### GRS suppressed cardiac hypertrophy and reduced pulmonary congestion in HFpEF mice

**Fig. 1:**
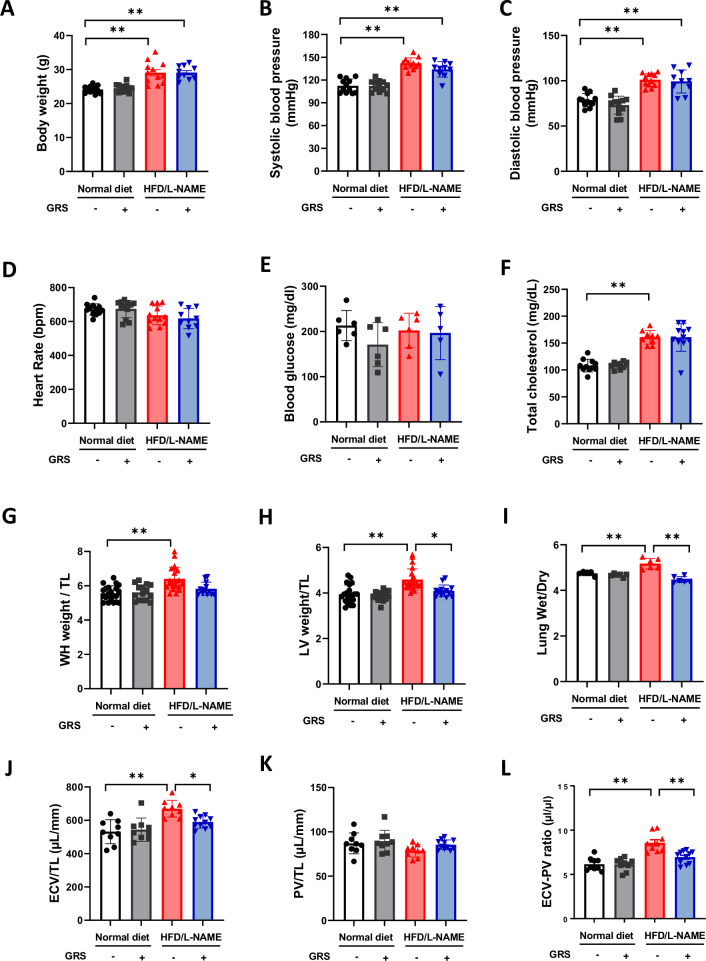
GRS suppressed cardiac hypertrophy and reduced pulmonary congestion in HFpEF mice. **A–F** Body weight, systolic and diastolic blood pressure, heart rate, blood glucose, and total cholesterol in mice fed normal diet, normal diet+GRS, HFD/L-NAME, or HFD/L-NAME + GRS for 5 weeks (*n* = 5–12). **G**, **H** Ratios of whole heart (WH) weight to tibial length (TL) and left ventricle (LV) weight to TL the indicated groups (*n* = 12–21). **I** Ratio of wet lung weight to dry lung weight in the indicated groups (*n* = 5–6). **J**,** K** Extracellular volume (ECV) and plasma volume (PV) adjusted for TL in the indicated groups (*n* = 9–11). **L** Ratio of ECV to PV in the indicated groups (*n* = 9–11). *P* < 0.05, **P < 0.01: post-hoc Tukey’s comparison test

We examined the effect of GRS on hemodynamic and metabolic parameters and organ weights in the HFpEF model. The body weight (BW) (Fig. 1A), blood pressure (Fig. 1B, C), and blood total cholesterol levels (Fig. 1F) increased after administration of HFD/L-NAME, but remained unaffected by GRS. Neither HFD/L-NAME nor GRS changed heart rate (Fig. 1D) or blood glucose levels (Fig. 1E). GRS suppressed HFD/L-NAME-induced increases in whole heart (WH) and left ventricle (LV) weights in addition to the wet/dry ratio of lung weight (Fig. 1G, H, I). Consistent with these changes, it decreased extracellular volume (ECV) without changes in plasma volume (PV) (Fig. 1J, K), resulting in decreases in ratio of ECV to PV (Fig. 1L) and interstitial fluid volume (IFV) in HFD/L-NAME-treated mice (Supplementary Fig. 2). These data suggest that GRS suppresses cardiac hypertrophy and lung and systemic congestion/edema in HFpEF mice.

### GRS ameliorated diastolic dysfunction and cardiomyocyte hypertrophy in HFpEF mice

**Fig. 2:**
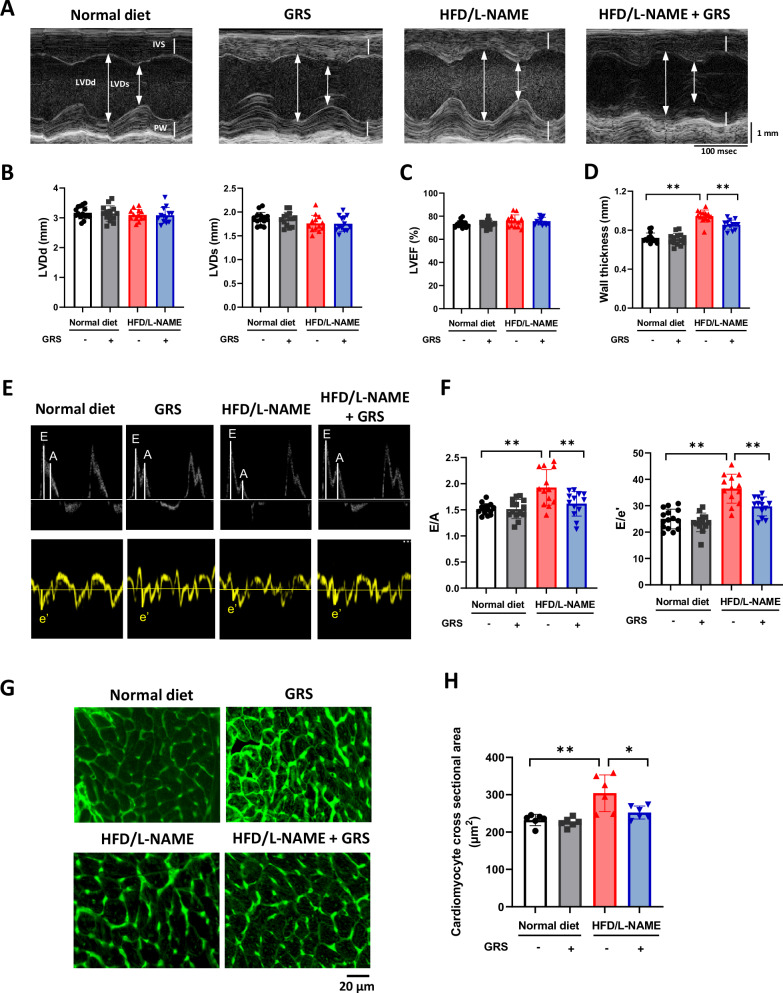
GRS ameliorated diastolic dysfunction and cardiomyocyte hypertrophy in HFpEF mice. **A** Representative echocardiographic images of mice fed normal diet, normal diet+GRS, HFD/L-NAME, or HFD/L-NAME + GRS for 5 weeks (*n* = 13–15). Long two-way arrows and short two-way arrows indicate left ventricular end-diastolic diameter (LVDd) and left ventricular end-systolic diameter (LVDs), respectively. **B–D** Quantitative analysis of LVDd, LVDs, LV ejection fraction (LVEF), and LV wall thickness evaluated by echocardiography at 5 weeks in the indicated groups (*n* = 13–15). **E**, **F** Representative echocardiographic images of LV inflow in the indicated groups. Quantitative analysis of ratios of E to A and E to e’ evaluated by echocardiography in the indicated groups (*n* = 13–15). **G**, **H** Representative images of wheat germ agglutinin (WGA)-stained heart sections in the indicated groups. Quantitative analysis of cardiomyocyte hypertrophy as assessed by cross-sectional area in the indicated groups (*n* = 6). Bar=20 μm. *P < 0.05, **P < 0.01: post-hoc Tukey’s comparison test

We investigated the effect of GRS on echocardiographic parameters. Neither HFD/L-NAME nor GRS affected LV diameters and LV ejection fraction (LVEF) (Fig. 2A–C). GRS suppressed HFD/L-NAME-induced increase in LV wall thickness (Fig. 2A, D). HFD/L-NAME increased E/A and E/e’ ratios, indices of diastolic dysfunction, and GRS significantly attenuated them (Fig. 2E, F).

We also evaluated the pathology of LV tissues to elucidate the effect of GRS on cardiac remodeling. Using wheat germ agglutinin (WGA) staining, we observed that HFD/L-NAME increased the cardiomyocyte cross sectional area, whereas GRS decreased it (Fig. 2G, H). In contrast, using Masson-trichrome staining, we observed that neither HFD/L-NAME nor GRS affected interstitial fibrosis (Supplementary Fig. 3).

### GRS alleviated phosphorylation of mitogen-activated protein kinases in HFpEF mouse hearts

**Fig. 3:**
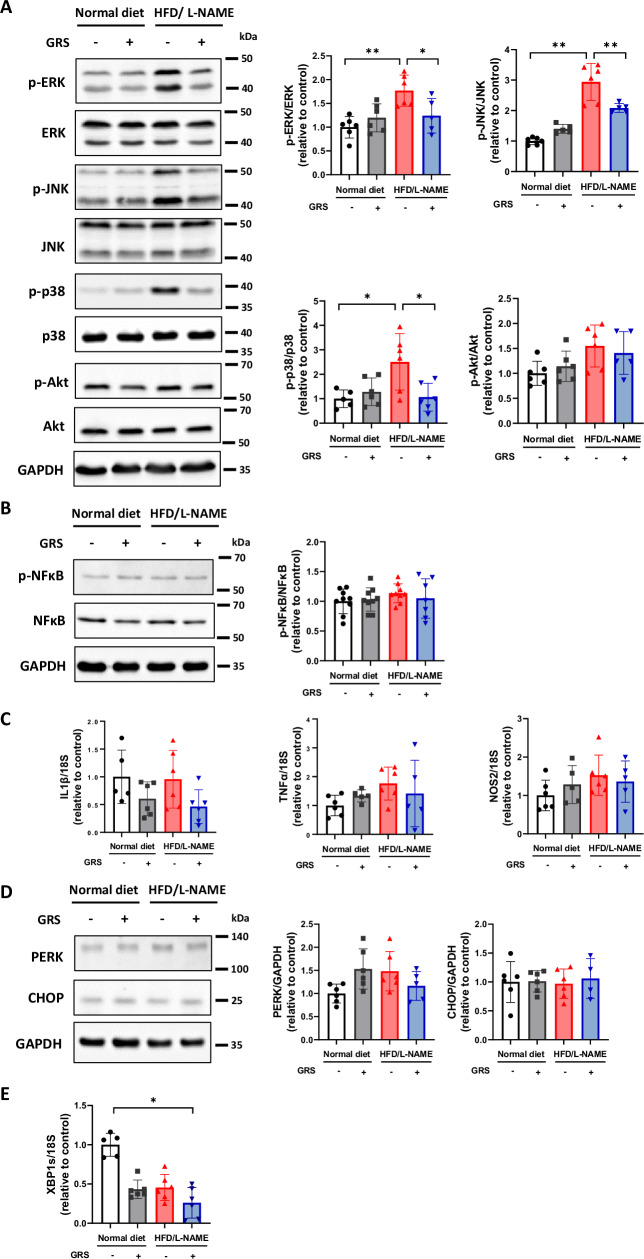
GRS alleviated phosphorylation of mitogen-activated protein kinases and did not affect inflammation- and endoplasmic reticulum stress-related factors in HFpEF. **A** Representative immunoblots and quantitative analysis of phosphorylated ERK, ERK, phosphorylated JNK, JNK, phosphorylated p38, p38, phosphorylated Akt, Akt, and GAPDH in the hearts of mice fed normal diet, normal diet+GRS, HFD/L-NAME, or HFD/L-NAME + GRS for 5 weeks. (*n* = 5–6). **B** Representative immunoblots and quantitative analysis of phosphorylated NF-κB, NF-κB, and GAPDH in the hearts of mice fed normal diet, normal diet+GRS, HFD/L-NAME, or HFD/L-NAME + GRS for 5 weeks (*n* = 7–9). **C** mRNA levels of *IL1b*, *TNFa*, and *NOS2* in the hearts of the indicated groups (*n* = 5–6). **D** Representative immunoblots and quantitative analysis of PERK, CHOP, and GAPDH in hearts of the indicated groups (*n* = 4–6). **E** mRNA levels of *XBP1s* in the hearts of the indicated groups (*n* = 5–6). *P* < 0.05, **P < 0.01: post-hoc Tukey’s comparison test

To elucidate the mechanism underlying the anti-hypertrophic effect of GRS, we assessed MAPKs such as ERK, JNK, and p38, and Akt, major hypertrophic mediators. HFD/L-NAME increased phosphorylation of ERK, JNK, and p38 in hearts and GRS suppressed them (Fig. 3A). In contrast, HFD/L-NAME tended to increase the Akt phosphorylation and GRS did not change it (Fig. 3A). These findings suggest that the suppression of MAPK activity is involved in the antihypertrophic effect of GRS.

Inflammation and endoplasmic reticulum (ER) stress are known to play pivotal roles in the development of HFpEF. Thus, we investigated the changes in these pathways in HFpEF mouse hearts. HFD/L-NAME did not affect NF-κB phosphorylation (Fig. 3B) and the mRNA levels of TNFα, IL-1β, and NOS2 and GRS had no effect on them (Fig. 3C). The protein levels of PERK and CHOP were unchanged in the heart treated with HFD/L-NAME and/or GRS (Fig. 3D). HFD/L-NAME tended to decrease XBP1 splicing variant, but GRS did not affect it (Fig. 3E).

### ICAT and β-catenin were involved in the protective effects of GRS against HFpEF mouse hearts

**Fig. 4:**
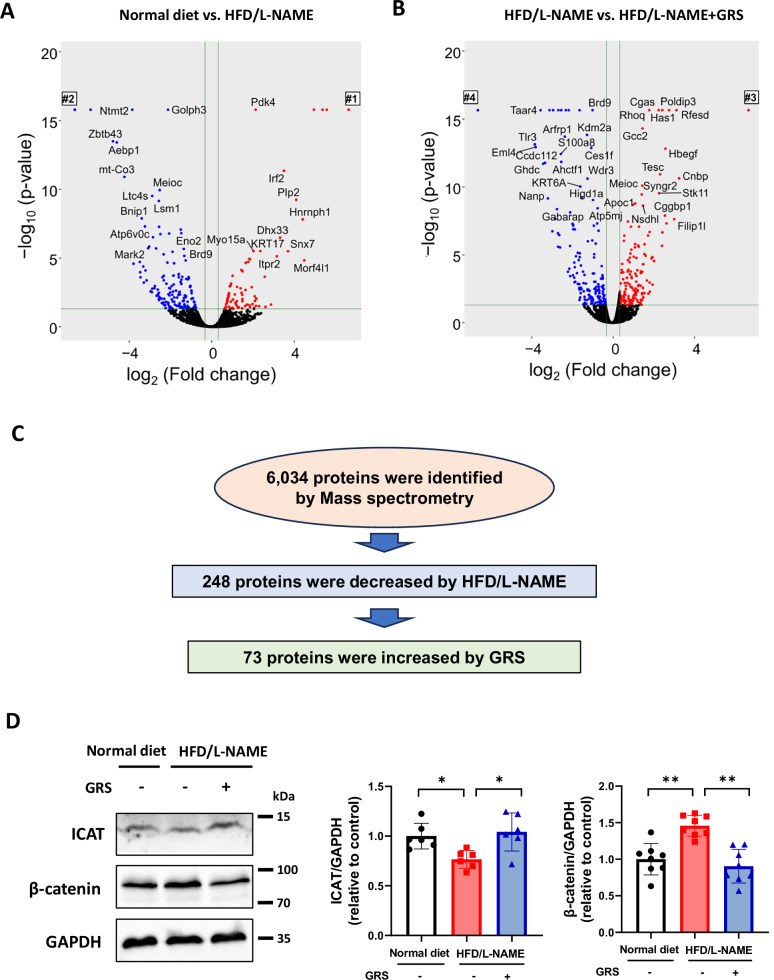
ICAT and β-catenin were involved in the protective effects of GRS against HFpEF hearts. **A** Volcano blots of the different expressed proteins (DEPs) in HFD/L-NAME to control. #1 indicates 199 proteins increased in HFD/L-NAME compared to control. #2 indicates 99 proteins decreased in HFD/L-NAME compared with control. **B** Volcano blots of DEPs in HFD/L-NAME + GRS to HFD/L-NAME. #3 indicates 91 proteins increased in HFD/L-NAME + GRS compared with HFD/L-NAME. #4 indicates 111 proteins decreased in HFD/L-NAME + GRS compared with HFD/L-NAME. **C** Selection of proteins decreased by HFD/L-NAME and increased by GRS. **D** Representative immunoblots and quantitative analysis of ICAT, β-catenin, and GAPDH in hearts of the indicated groups (*n* = 5–8). *P < 0.05, **P < 0.01: post-hoc Tukey’s comparison test

To further elucidate the molecular mechanism mediating the effect of GRS, we performed proteomics analysis using LC-MS/MS and detected 6034 proteins in the heart. HFD/L-NAME increased 277 proteins and decreased 248 proteins in the heart (Fig. 4A). GRS increased 247 proteins and decreased 273 proteins in the heart treated with HFD/L-NAME (Fig. 4B). By integrating these data, we found that HFD/L-NAME decreased and GRS increased 73 proteins (Fig. 4C). Among them, inhibitor of β-catenin and T-cell factor (ICAT) is known to suppress Wnt/β-catenin.signaling [31, 32], which plays an important role in cardiac hypertrophy [33–35]. Immunoblotting was used to assess protein levels to confirm the change in this signaling. HFD/L-NAME decreased ICAT and increased β-catenin and GRS prevented these changes (Fig. 4D).

Transforming growth factor-β1 (TGF-β1)/SMAD axis is known to be involved in inflammation [36]. In mass spectrometry analysis, TGF-β1 was not detected in any of the four groups. Regarding SMAD family, SMAD1, 2, 4, and 6 were detected. Among them, SMAD4 was decreased in HFpEF hearts compared with control hearts, whereas, GRS did not change it (Supplementary Table 1). On the other hand, SMAD1 was increased in HFpEF hearts compared with control hearts and GRS decreased it (Supplementary Table 1).

In GO analysis, cytoplasm- and ribosome-related factors were enriched in HFpEF (Supplementary Fig. 4A). On the other hand, ribosome- and mitochondria-related factors were enriched by GRS (Supplementary Fig. 4B). We further investigated the mitochondrial protein profiles. Among mitochondria-related proteins, we can detect 9 proteins which were significantly changed by GRS as shown below: ATP synthase subunit e, mitochondrial, ATP synthase subunit a, NADH-ubiquinone oxidoreductase chain 3, NADH-ubiquinone oxidoreductase chain 4, ATP synthase F(0) complex subunit B1, mitochondrial, ATP synthase subunit epsilon, mitochondrial, NADH dehydrogenase [ubiquinone] iron-sulfur protein 6, mitochondrial, NADH dehydrogenase [ubiquinone] 1 subunit C2, and NADH dehydrogenase [ubiquinone] iron-sulfur protein 4, mitochondrial (Supplementary Table 2).

### GRS suppressed ISO-induced increases in β-catenin and cardiomyocyte hypertrophy

**Fig. 5:**
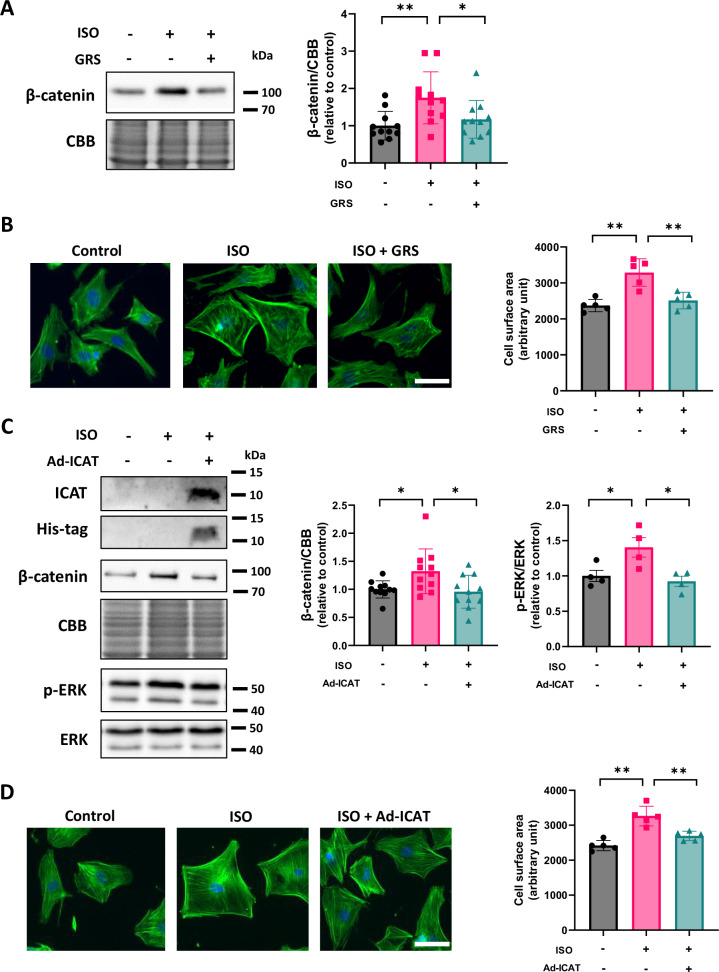
GRS suppressed ISO-induced increases in β-catenin and cardiomyocyte hypertrophy. **A** Representative immunoblots and quantitative analysis of β-catenin and CBB in NRVMs treated with or without GRS (100 μg/mL) in the presence or absence of ISO (10 μM) for 48 hours (*n* = 11). **B** Cell surface area of NRVMs treated with or without GRS (100 μg/mL) in the presence or absence of ISO (10 μM) for 48 hours (*n* = 5). A minimum of 100 cardiomyocytes from each culture dish was measured. Scale bar, 50 µm. **C** Representative immunoblots and quantitative analysis of ICAT, His-Tag, β-catenin, phosphorylated ERK, ERK, and CBB in NRVMs treated with or without adenovirus harboring ICAT (1 MOI) in the presence or absence of ISO (10 μM) for 48 hours (β-catenin: *n* = 11, phosphorylated ERK: *n* = 4). **D** Cell surface area of NRVMs treated with or without adenovirus harboring ICAT (1 MOI) in the presence or absence of ISO (10 μM) for 48 h (*n* = 5). A minimum of 100 cardiomyocytes from each culture dish was measured. Scale bar, 50 µm. *p < 0.05, **p < 0.01: post hoc Tukey’s comparison test

We assessed the direct effect of GRS on cardiomyocyte hypertrophy in in vitro experiments. GRS (100 μg/ml, 48 h) suppressed ISO (10 µM, 48 h)-induced increases in β-catenin in cardiomyocytes (Fig. 5A). Consistently, it prevented ISO-induced increases in cardiomyocyte surface area (Fig. 5B). These data indicate that GRS directly attenuates cardiomyocyte hypertrophy by suppressing β-catenin. To investigate whether the anti-hypertrophic effect of GRS is mediated by ICAT, we evaluated ICAT protein levels. However, we could not detect ICAT in cardiomyocytes by immunoblot. Thus, we conducted overexpression of His-tagged ICAT in cardiomyocytes. His protein was detected by immunoblot (Fig. 5C), indicating ICAT protein was definitely overexpress in cardiomyocytes. Overexpression of ICAT suppressed ISO-induced increases in β-catenin and phosphorylated ERK in cardiomyocytes (Fig. 5C), accompanied by a decrease in cardiomyocyte surface area (Fig. 5D). Taken together, ICAT negatively regulates cardiomyocyte hypertrophy by suppressing β-catenin and ERK.

### Cinnamaldehyde and Alisol B 23-acetate suppressed ISO-induced increases in β-catenin and cardiomyocyte hypertrophy

**Fig. 6:**
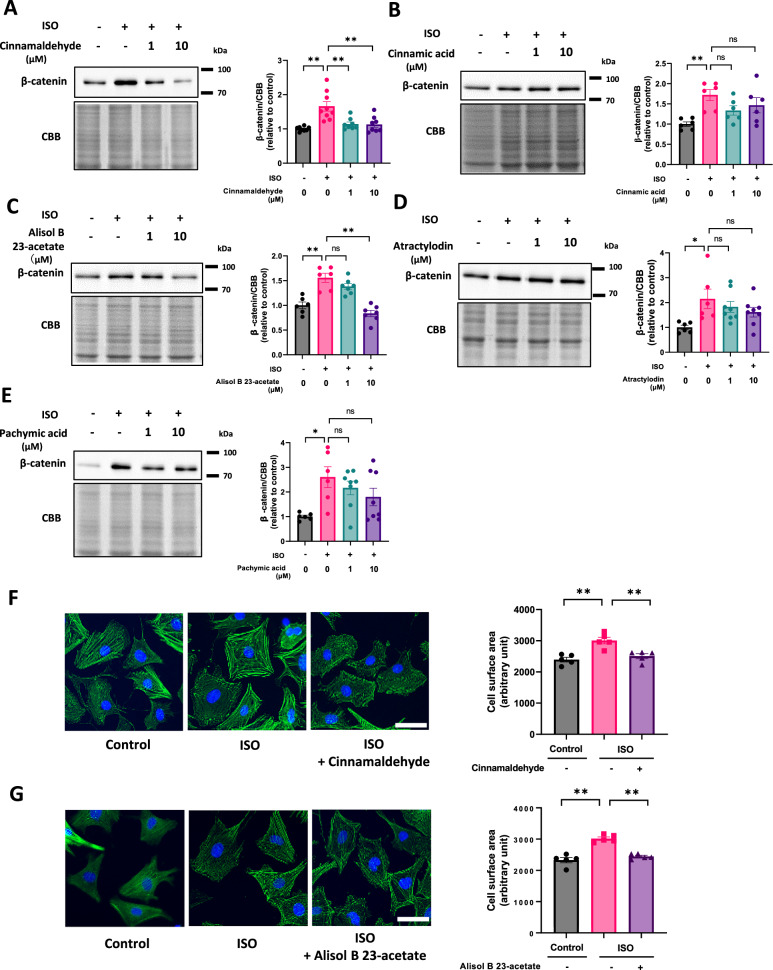
Cinnamaldehyde and Alisol B 23-acetate suppressed ISO-induced increases in β-catenin and cardiomyocyte hypertrophy. **A–E** Representative immunoblots and quantitative analyses of β-catenin and CBB in NRVMs treated with or without cinnamaldehyde (**A**), cinnamic acid (**B**), alisol B 23-acetate (**C**), atractylodin (**D**), or pachymic acid (**E**) at concentrations of 1–10 µM, in the presence or absence of ISO (10 μM) for 48 h (*n* = 6–9). **F**, **G** Cell surface area of NRVMs treated with or without cinnamaldehyde (10 µM) or alisol B 23-acetate (10 µM), in the presence or absence of ISO (10 µM) for 48 h (*n* = 5). A minimum of 100 cardiomyocytes per culture dish were measured. Scale bar, 50 µm. *p < 0.05, **p < 0.01; post hoc Tukey’s test

To elucidate which ingredient of GRS exerts the anti-hypertrophic effect, we evaluated the suppressive effect of several GRS ingredients on β-catenin, such as cinnamaldehyde, cinnamic acid, alisol B 23-acetate, atractylodin, pachymic acid. Among them, cinnamaldehyde (1 µM and 10 µM) and alisol B 23-acetate (10 µM) suppressed β-catenin in ISO-treated cardiomyocytes (Fig. 6A, C). On the other hand, neither cinnamic acid, atractylodin, nor pachymic acid did so (Fig. 6B, D, E). Consistent with these changes, cinnamaldehyde (10 µM) or alisol B 23-acetate (10 µM) attenuated ISO-induced cardiomyocyte hypertrophy (Fig. 6F, G). These data suggest that cinnamaldehyde and alisol B 23-acetate are potential ingredients exerting anti-hypertrophic effects of GRS.

## Discussion

In this study, we demonstrated that GRS attenuated cardiac hypertrophy, diastolic dysfunction, and pulmonary congestion/edema in HFpEF without changes in blood pressure. GRS suppressed not only MAPKs but also β-catenin, a positive regulator of cardiac hypertrophy, and preserved ICAT, a negative regulator of cardiac hypertrophy, in HFpEF hearts. Among GRS ingredients, cinnamaldehyde and alisol B 23-acetate directly suppressed ISO-induced increases in β-catenin in cardiomyocytes. Our data provide a potential anti-hypertrophic effect of GRS via ICAT-β-catenin/ERK axis.

GRS, an herbal formula containing five components, has been traditionally used to for the treatment of fluid regulation disorders in Asian countries, including Japan, China, and Korea [12]. GRS is effective for decongestion in patients with HF [15, 16]. The first large-scale randomized pragmatic trial to evaluate the efficacy and safety of GRS in patients with HF and volume overload is currently underway [22]. In this study, GRS attenuated HFD/L-NAME-induced cardiac hypertrophy and diastolic dysfunction (Fig. 2). GRS is known to have water diuretic effects similar to tolvaptan. We cannot exclude the possibility that water diuretic effects mediate the beneficial impact of GRS on HFpEF. However, notably, GRS decreased lung wet/dry ratio in HFpEF without changes in BW and blood pressure (Fig. 1). This cannot be explained solely by the diuretic effect. These findings indicate that improved diastolic function leads to decreased pulmonary congestion/edema.

Mechanistically, GRS suppressed phosphorylation of ERK, JNK, and p38 in HFpEF hearts (Fig. 3). Numerous studies have shown that these MAPKs are master regulators of cardiac hypertrophy [37–40]. We speculate that suppression of ERK, JNK, and p38 may mediate the beneficial effects of GRS on cardiac hypertrophy and diastolic dysfunction in HFpEF. Furthermore, comprehensive mass spectrometric analysis demonstrated that HFD/L-NAME decreased ICAT and GRS preserved it in HFpEF hearts (Fig. 4). ICAT has been reported to negatively regulate β-catenin protein levels and Wnt/β-catenin signaling by interacting with it [31, 32]. Recent several studies have revealed that Wnt/β-catenin signaling plays a critical role in the development of cardiac hypertrophy [33–35]. In addition, Wnt/β-catenin signaling is known to be a specific pathway associated with HFpEF [41]. We found that overexpression of ICAT attenuated cardiomyocyte hypertrophy by suppressing β-catenin and ERK (Figs. 5C and 5D). Intriguingly, β-catenin and ERK make positive feedback loop [42]. Our data suggest that ICAT is a critical regulator of β-catenin and ERK and ICAT-β-catenin/ERK axis is a potential novel therapeutic target in cardiac hypertrophy and HFpEF. GO analysis demonstrated that ribosome-related factors were enriched in HFpEF and that mitochondria- and ribosome-related factors were enriched by GRS (Supplementary Fig. 4). Wnt/β-catenin signaling is known to be involved in ribosome and mitochondrial biogenesis [43, 44]. In fact, we could detect 9 proteins which were significantly changed by GRS (Supplementary Table 2). Therefore, the beneficial effects of GRS on HFpEF pathophysiology may also be due to subsequent changes in factors related to mitochondria- and ribosome following Wnt/β-catenin signaling.

Inflammation and ER stress are intimately involved in the development of HFpEF [9, 45]. However, in this study, the phosphorylated NF-κB levels and mRNA levels of TNFα, IL-1β, and IL-6 were not increased in HFpEF (Fig. 3B, C). The short-term administration of HFD/L-NAME may not induce sufficient inflammation. TGF-β1/SMAD axis is known to be involved in inflammation [36]. Among TGF-β1 and SMAD family, only SMAD1 was increased in HFpEF hearts and GRS decreased it (Supplementary Table 1). SMAD1 might be involved in pathophysiology of HFpEF and beneficial effects of GRS. However, in this study, inflammation was not evident in HFpEF. Thus, further study is needed to elucidate the precise role of TGF-β1/SMAD axis in the beneficial effects of GRS.

With regard with ER stress, neither HFD/L-NAME nor GRS altered CHOP and PERK (Fig. 3D). A previous study has shown that a decrease in XBP1s mediates diastolic dysfunction in HFD/L-NAME-treated mice [9]. However, GRS did not increase XBP1s in HFpEF (Fig. 4E). These findings indicate that GRS ameliorates diastolic dysfunction independently of inflammation and ER stress.

GRS suppressed ISO-induced increases in β-catenin and attenuated cardiomyocyte hypertrophy in in vitro experiments (Fig. 5A, B), indicating that, at least, the anti-hypertrophic effect is due to the direct action of GRS. Several previous studies demonstrated that cinnamaldehyde, a GRS component, alleviated pressure overload- or phenylephrine-induced cardiac hypertrophy [17–19]. In this study, we found that, among several ingredients of GRS, cinnamaldehyde and alisol B 23-acetate, but not cinnamic acid, atractylodin, or pachymic acid, suppressed β-catenin protein levels in ISO-treated cardiomyocytes (Fig. 6A–E). Cinnamaldehyde and alisol B 23-acetate also attenuated ISO-induced cardiomyocyte hypertrophy (Fig. 6F, G). Thus, the antihypertrophic effect of GRS is thought to be primarily due to these ingredients.

There are various mouse models of HFpEF, including obesity/high-fat diet models, mild TAC models, hypertension models and catecholamine-treated models. Among these models, HFD/L-NAME model nicely mimics phenotypes of HFpEF in human in terms of diastolic dysfunction. In addition, ERK is a key player in the pathophysiology of cardiac hypertrophy in response to pressure overload [46]. β-catenin critically mediates angiotensin II-induced cardiac hypertrophy [47]. Thus, GRS might be beneficial for other HFpEF models except for HFD/L-NAME model.

Several traditional herbal formulations, particularly those containing Glycyrrhiza, cause hypokalemia due to sodium retention and accelerated potassium excretion, which is a side effect known as pseudoaldosteronism [48]. GRS does not contain glycyrrhiza and did not affect blood and urine sodium or potassium balance in this study (data not shown). GRS is likely to be safe to use in patients with HF without concern for pseudoaldosteronism.

In conclusion, GRS attenuates cardiac hypertrophy and diastolic dysfunction as well as pulmonary congestion/edema in HFpEF. A novel ICAT-β-catenin/ERK axis is involved in the cardioprotective effects of GRS. GRS is a potential therapeutic herbal formula against HFpEF.

## Supplementary information

Supplementary table

Supplementary figure

Supplementary figure legends
