# Supplementary material for: Goreisan attenuates cardiac hypertrophy and diastolic dysfunction in heart failure with preserved ejection fraction induced by HFD/L-NAME via regulation of ICAT-β-catenin/ERK axis
Source: Hypertens Res. 2025 Sep 3;48(11):2882–96. doi: 10.1038/s41440-025-02348-z (PMC12586149; doi:10.1038/s41440-025-02348-z)
Supplement: Supplementary file 1 — Supplementary table [file 41440_2025_2348_MOESM1_ESM.docx]

**Supplementary Table 1**

| **UniProtID** | **Description** | **Gene Symbol** | **HFD/L-NAME vs Normal** | | **HFD/L-NAME+GRS vs HFD/L-NAME** | |
| --- | --- | --- | --- | --- | --- | --- |
|  |  |  | **Ratio** | **Adjusted P-Value** | **Ratio** | **Adjusted P-Value** |
| P70340 | Mothers against decapentaplegic homolog 1 | Smad1 | 0.01 | <0.00001 | 100.000 | <0.00001 |
| Q62432 | Mothers against decapentaplegic homolog 2 | Smad2 | 1.514 | 0.71057 | 1.013 | 0.981319204 |
| P97471 | Mothers against decapentaplegic homolog 4 | Smad4 | 0.148 | 0.00108 | 1.612 | 0.460067903 |
| O35182 | Mothers against decapentaplegic homolog 6 | Smad6 | 1.798 | 0.56179 | 0.865 | 0.833454937 |

**Supplementary Table 2**

| **UniProtID** | **Description** | **Gene Symbol** | **HFD/L-NAME vs Normal** | | **HFD/L-NAME+GRS vs HFD/L-NAME** | |
| --- | --- | --- | --- | --- | --- | --- |
|  |  |  | **Ratio** | **Adjusted P-Value** | **Ratio** | **Adjusted P-Value** |
| Q06185 | ATP synthase subunit e, mitochondrial | Atp5me | 1.045 | 0.93316 | 1.445 | <0.00001 |
| P00848 | ATP synthase subunit a | Mtatp6 | 0.568 | 0.05599 | 0.552 | <0.00001 |
| P03899 | NADH-ubiquinone oxidoreductase chain 3 | mt-Nd3 | 0.417 | 0.00001 | 1.463 | <0.00001 |
| P03911 | NADH-ubiquinone oxidoreductase chain 4 | Mtnd4 | 0.947 | 0.99210 | 0.760 | 0.02458 |
| Q9CQQ7 | ATP synthase F(0) complex subunit B1, mitochondrial | Atp5pb | 0.871 | 0.90466 | 0.769 | 0.03853 |
| P56382 | ATP synthase subunit epsilon, mitochondrial | Atp5f1e | 0.649 | 0.16930 | 0.516 | <0.00001 |
| P52503 | NADH dehydrogenase [ubiquinone] iron-sulfur protein 6, mitochondrial | Ndufs6 | 0.671 | 0.21763 | 0.759 | 0.02394 |
| Q9CQ54 | NADH dehydrogenase [ubiquinone] 1 subunit C2 | Ndufc2 | 0.657 | 0.17069 | 0.764 | 0.02994 |
| Q9CXZ1 | NADH dehydrogenase [ubiquinone] iron-sulfur protein 4, mitochondrial | Ndufs4 | 0.508 | 0.00246 | 0.693 | 0.00031 |
