# Supplementary material for: Goreisan attenuates cardiac hypertrophy and diastolic dysfunction in heart failure with preserved ejection fraction induced by HFD/L-NAME via regulation of ICAT-β-catenin/ERK axis
Source: Hypertens Res. 2025 Sep 3;48(11):2882–96. doi: 10.1038/s41440-025-02348-z (PMC12586149; doi:10.1038/s41440-025-02348-z)
Supplement: Supplementary file 2 — Supplementary figure [file 41440_2025_2348_MOESM2_ESM.pdf]

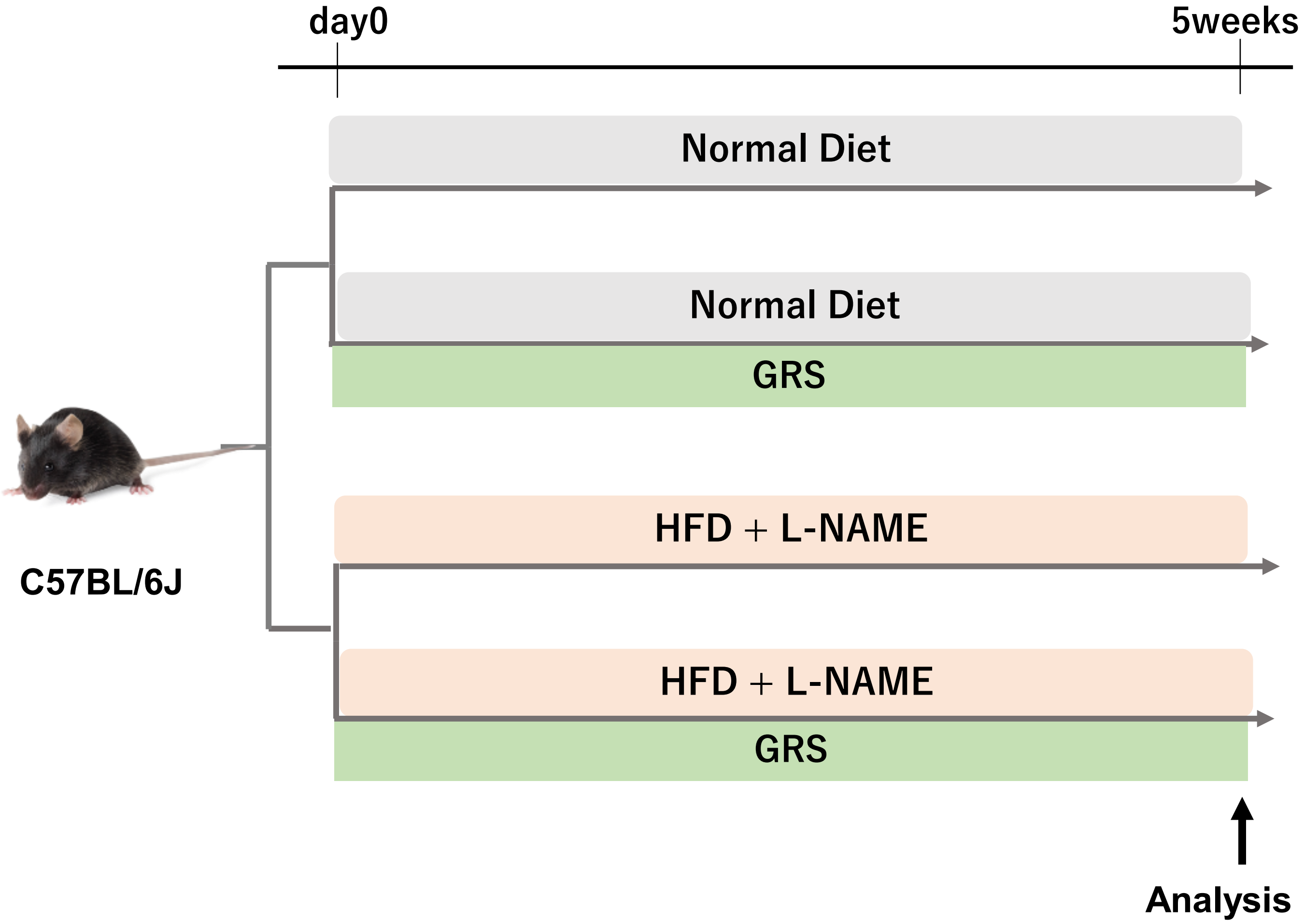

HFD (High fat diet)：60% fat
L-NAME (N-nitro-L-arginine methylester)：0.5 g/L in drinking water
GRS (Goreisan)：5.9 mg/kcal

Supplementary Figure 1. Experimental protocol in this study

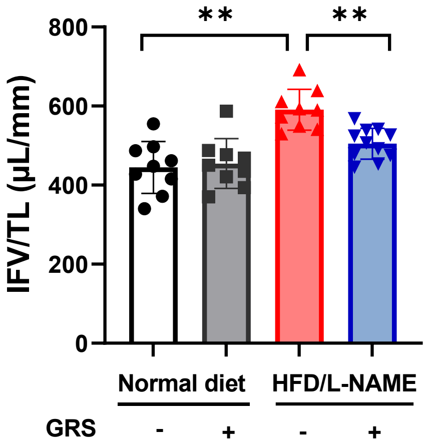

Supplementary Figure 2. GRS reduced IFV in HFpEF

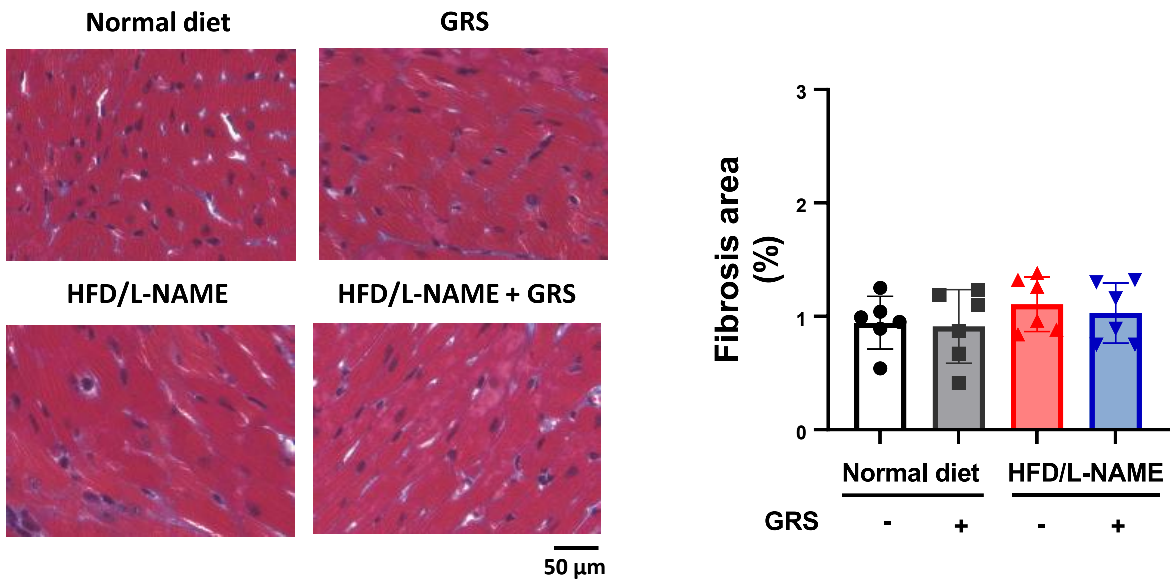

Supplementary Figure 3. GRS did not change interstitial fibrosis in HFpEF mice

A

HFD/L-NAME vs. Normal diet

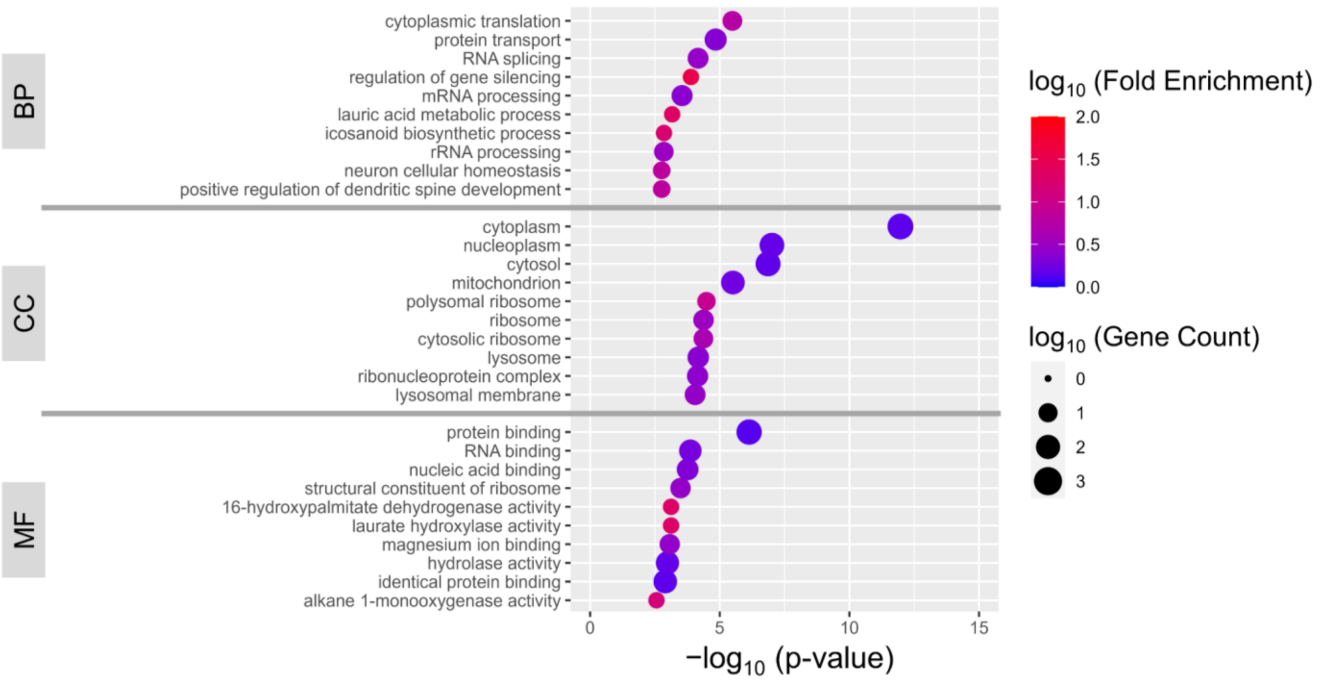

B

HFD/L-NAME+GRS vs. HFD/L-NAME

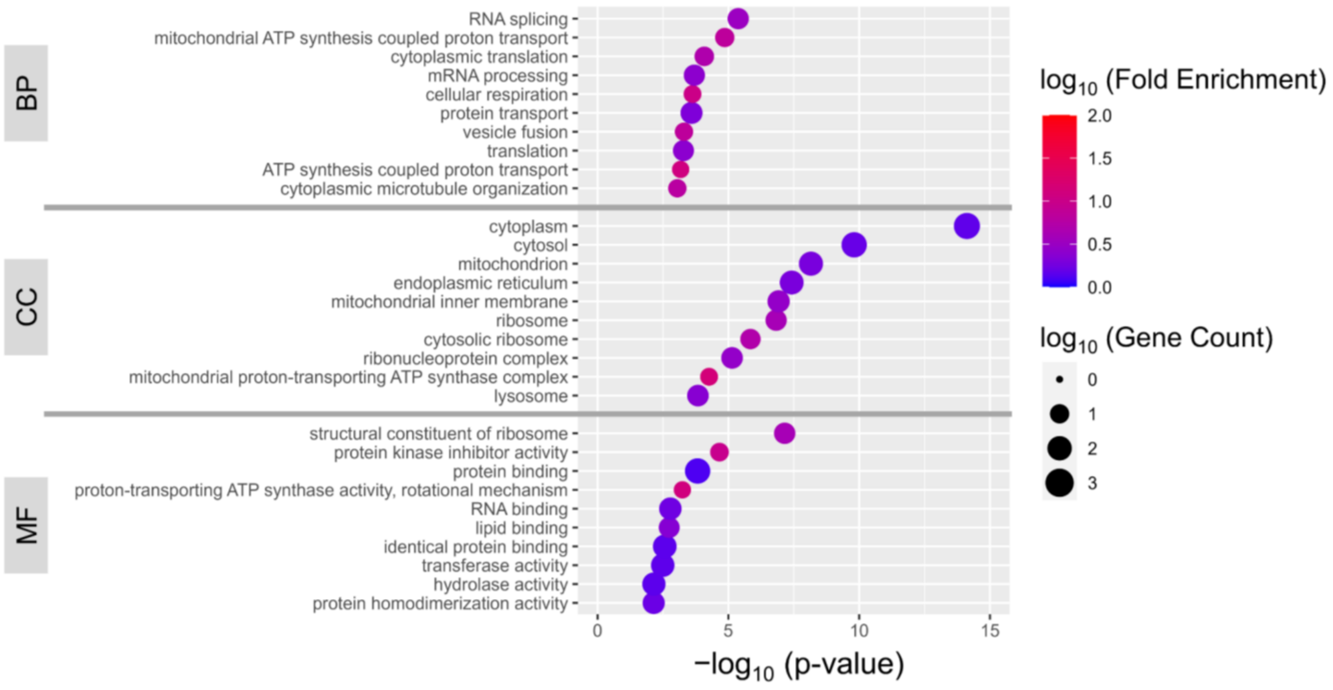

Supplementary Figure 4. GO enrichment analyses using DAVID database for DEPs in HFpEF hearts
