# Supplementary material for: Goreisan attenuates cardiac hypertrophy and diastolic dysfunction in heart failure with preserved ejection fraction induced by HFD/L-NAME via regulation of ICAT-β-catenin/ERK axis
Source: Hypertens Res. 2025 Sep 3;48(11):2882–96. doi: 10.1038/s41440-025-02348-z (PMC12586149; doi:10.1038/s41440-025-02348-z)
Supplement: Supplementary file 3 — Supplementary figure legends [file 41440_2025_2348_MOESM3_ESM.docx]

**Supplementary figure legends**

**Supplementary Figure 1. Experimental protocol in this study.** Mice were divided into four treatment groups and exposed to one of the following regimens: normal diet (control); normal diet + GRS (5.9 mg/kcal); HFD (60% fat) with L-NAME (0.5 g/L in drinking water); HFD + GRS with L-NAME for 5 weeks.

**Supplementary Figure 2. GRS reduced IFV in HFpEF.** Interstitial fluid volume (IFV) in mice fed normal diet, normal diet+GRS, HFD/L-NAME, or HFD/L-NAME+GRS for 5 weeks (n=9-11). *P<0.05, **P<0.01: post-hoc Tukey’s comparison test.

**Supplementary Figure 3.** **GRS did not change interstitial fibrosis in HFpEF mice.** Representative images of MT-stained heart sections in mice fed normal diet, normal diet+GRS, HFD/L-NAME, or HFD/L-NAME+GRS for 5 weeks. Interstitial fibrosis as assessed by collagen volume in the indicated groups (n=6). Bar=20μm. *P<0.05, **P<0.01: post-hoc Tukey’s comparison test.

**Supplementary Figure 4.** **GO enrichment analyses using DAVID database for DEPs in HFpEF hearts.** The bottom 10 p-values of t-test in biological process (BP), cellular component (CC), and molecular function (MF), respectively. **(A)** Analysis of the different expressed proteins (DEPs) in HFD/L-NAME compared with normal diet (control). **(B)** Analysis of DEPs in HFD/L-NAME+GRS to HFD/L-NAME.
